# Dipeptidyl peptidase-4 inhibition prevents nonalcoholic steatohepatitis–associated liver fibrosis and tumor development in mice independently of its anti-diabetic effects

**DOI:** 10.1038/s41598-020-57935-6

**Published:** 2020-01-22

**Authors:** Mitsuhiro Kawakubo, Miyako Tanaka, Kozue Ochi, Akiko Watanabe, Marie Saka-Tanaka, Yohei Kanamori, Naoki Yoshioka, Satoko Yamashita, Moritaka Goto, Michiko Itoh, Ibuki Shirakawa, Sayaka Kanai, Hiromi Suzuki, Makoto Sawada, Ayaka Ito, Masatoshi Ishigami, Mitsuhiro Fujishiro, Hiroshi Arima, Yoshihiro Ogawa, Takayoshi Suganami

**Affiliations:** 1 Department of Molecular Medicine and Metabolism, Research Institute of Environmental Medicine, Nagoya University, Nagoya, Japan; 2 Department of Brain Function, Research Institute of Environmental Medicine, Nagoya University, Nagoya, Japan; 3 Department of Endocrinology and Diabetes, Nagoya University Graduate School of Medicine, Nagoya, Japan; 4 Department of Immunometabolism, Nagoya University Graduate School of Medicine, Nagoya, Japan; 5 Department of Nephrology, Nagoya University Graduate School of Medicine, Nagoya, Japan; 6 Department of Gastroenterology and Hepatology, Nagoya University Graduate School of Medicine, Nagoya, Japan; 7 Department of Molecular Pharmacokinetics, Nagoya University Graduate School of Medicine, Nagoya, Japan; 8 Pharmaceutical Research Laboratories, Sanwa Kagaku Kenkyusho Co., Ltd., Nagoya, Japan; 9 Department of Organ Network and Metabolism, Graduate School of Medical and Dental Sciences, Tokyo Medical and Dental University, Tokyo, Japan; 10 Kanagawa Institute of Industrial Science and Technology, Kawasaki, Japan; 11 Department of Molecular Endocrinology and Metabolism, Graduate School of Medical and Dental Sciences, Tokyo Medical and Dental University, Tokyo, Japan; 12 Department of Medicine and Bioregulatory Science, Graduate School of Medical Sciences, Kyushu University, Fukuoka, Japan; 13 Japan Agency for Medical Research and Development, CREST, Tokyo, Japan

**Keywords:** Cell death and immune response, Chronic inflammation, Diabetes complications, Non-alcoholic steatohepatitis

## Abstract

Nonalcoholic steatohepatitis (NASH) is a hepatic phenotype of the metabolic syndrome, and increases the risk of cirrhosis and hepatocellular carcinoma (HCC). Although increasing evidence points to the therapeutic implications of certain types of anti-diabetic agents in NASH, it remains to be elucidated whether their effects on NASH are independent of their effects on diabetes. Genetically obese melanocortin 4 receptor–deficient (MC4R-KO) mice fed Western diet are a murine model that sequentially develops hepatic steatosis, NASH, and HCC in the presence of obesity and insulin resistance. In this study, we investigated the effect of the dipeptidyl peptidase-4 (DPP-4) inhibitor anagliptin on NASH and HCC development in MC4R-KO mice. Anagliptin treatment effectively prevented inflammation, fibrosis, and carcinogenesis in the liver of MC4R-KO mice. Interestingly, anagliptin only marginally affected body weight, systemic glucose and lipid metabolism, and hepatic steatosis. Histological data and gene expression analysis suggest that anagliptin treatment targets macrophage activation in the liver during the progression from simple steatosis to NASH. As a molecular mechanism underlying anagliptin action, we showed that glucagon-like peptide-1 suppressed proinflammatory and profibrotic phenotypes of macrophages *in vitro*. This study highlights the glucose metabolism–independent effects of anagliptin on NASH and HCC development.

## Introduction

Nonalcoholic steatohepatitis (NASH) is a progressive form of non-alcoholic fatty liver disease (NAFLD), and increases the risk of cirrhosis and hepatocellular carcinoma (HCC). Although the prevalence of NASH is rising in parallel with the global obesity pandemic, effective therapeutic strategies are still under development. Given that NASH is associated with obesity and diabetes, pharmacological interventions for metabolic derangements may also useful for treating NASH. In fact, mounting evidence suggests that certain types of anti-diabetic agents, such as inhibitors of sodium glucose cotransporter 2 (SGLT2) and dipeptidyl peptidase-4 (DPP-4), effectively ameliorate histologic parameters of NASH in mice and humans^1–3^. However, their long-term efficacy and safety, as well as their preventive effects on NASH-associated carcinogenesis, have not been verified.

DPP-4 is a serine protease that cleaves the N-terminal dipeptides of various hormones and cytokines in the circulation. DPP-4 inhibitors are commonly used to treat type 2 diabetes, because they enhance circulating levels of incretins, such as glucagon-like peptide-1 (GLP-1) and glucose-dependent insulinotropic polypeptide (GIP), to enhance insulin secretion^4^. In addition, several lines of evidence suggest the pleiotropic effects for DPP-4 inhibitors on the cardiovascular system and NAFLD, although the underlying mechanisms remain unclear^5,6^. Indeed, DPP-4 is ubiquitously expressed and acts on a variety of physiological substances^7^. DPP-4 expression is also increased in the liver of patients with hepatic steatosis^8^. Accordingly, it is important to elucidate whether metabolic improvement is necessary for the preventive effect of DPP-4 inhibitors on NASH.

To date, various animal models have been proposed for NASH using hepatotoxic agents, genetic engineering, and dietary challenges^9^, whereas few of them exhibit both obesity phenotypes and liver carcinogenesis. In this regard, we previously reported that genetically obese melanocortin 4 receptor–deficient (MC4R-KO) mice fed Western diet (WD) can act as a novel murine model that sequentially develops hepatic steatosis, NASH, and HCC in the presence of obesity and insulin resistance^10^. Using this model, we identified a unique histological structure termed “hepatic crown-like structures (hCLS)”, in which CD11c-positive macrophages aggregate around dead hepatocytes with large lipid droplets, thereby functioning as drivers of liver fibrosis^11,12^. In other words, hCLS provides a histological feature where hepatocyte death, a hallmark of NASH, triggers macrophage activation and liver fibrosis^11^. In particular, hCLS has been observed in patients with NASH and animal models, including dietary deficiencies of methionine and choline, and long-term feeding with WD^12^. These observations led us to investigate the effect of the DPP-4 inhibitor anagliptin on NASH and HCC development.

In this study, we found that treatment with anagliptin effectively prevented inflammation, fibrosis, and carcinogenesis in the liver of MC4R-KO mice fed WD, while only marginally affecting body weight, systemic glucose and lipid metabolism, and hepatic steatosis. Interestingly, anagliptin treatment suppressed the increase in the number of hCLS without affecting hepatic steatosis, suggesting that macrophages are an *in vivo* target of anagliptin. Our *in vitro* data showed that exendin-4, a GLP-1 receptor agonist, suppressed the proinflammatory and profibrotic phenotypes of cultured macrophages. This study demonstrates that anagliptin has preventive effects on the development of liver fibrosis and carcinogenesis, independent of the drug’s effects on systemic glucose and lipid metabolism, in a murine model of NASH.

## Results

### Anagliptin ameliorates NASH-like liver phenotypes in MC4R-KO mice

**Figure 1:**
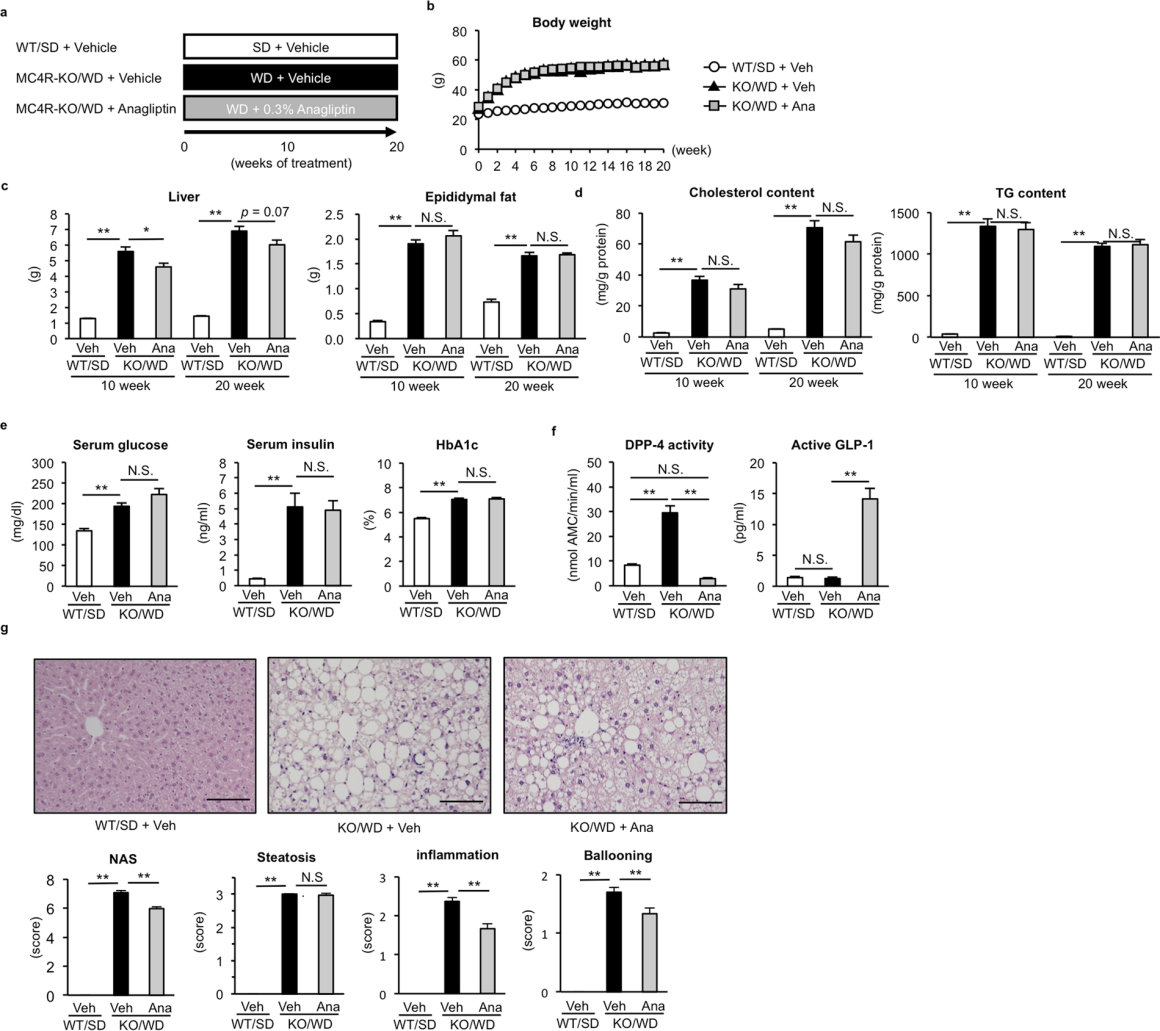
Anagliptin ameliorates NASH-like liver phenotypes in MC4R-KO mice. (**a**) Experimental protocol for examination of the preventive effect of anagliptin (Ana) on the development of NASH in melanocortin 4 receptor-deficient (MC4R-KO) mice fed Western diet (KO/WD). Wildtype (WT) mice on standard diet (WT/SD) were used as the control group. **(b)** Growth curve of WT and MC4R-KO mice. Open circle, WT/SD treated with the vehicle (*n* = 6); closed triangle, MC4R-KO/WD treated with the vehicle (*n* = 15); gray square, MC4R-KO/WD treated with anagliptin (*n* = 15), respectively. **(c)** Tissue weights of liver and epididymal fat after 10 and 20 weeks of treatment with anagliptin. **(d)** Liver cholesterol and triglyceride (TG) contents at 10 and 20 weeks. **(e)** Effect of anagliptin treatment on glucose metabolism at 10 weeks. **(f)** Effect of anagliptin treatment on the dipeptidyl peptidase-4 (DPP-4) activity and the active GLP-1 concentrations in the plasma at 10 weeks. **(g)** Hematoxylin and eosin staining of the liver at 20 weeks. Histological analysis using NASH activity score (NAS). *p < 0.05, **p < 0.01; N.S. not significant. WT/SD treated with vehicle (*n* = 6), KO/WD treated with anagliptin or vehicle (*n* = 12 and 15 at 10 and 20 weeks, respectively).

We previously reported that wildtype mice fed WD diet and MC4R-KO mice fed standard diet (SD) showed only simple hepatic steatosis^10^, and it has been reported that DPP-4 inhibitors ameliorate diet-induced hepatic steatosis in wildtype mice^13,14^. Thus, we focused on the effect of anagliptin on the NASH-associated liver fibrosis and tumor development. First, we determined the effect of anagliptin on the development of NASH-like liver phenotypes in MC4R-KO mice. Wildtype mice were fed SD and MC4R-KO mice were fed WD with or without anagliptin treatment (Ana or Veh) for up to 20 weeks (Fig. 1a). As previously shown^10^, MC4R-KO mice fed WD exhibited morbid obesity with dysregulated glucose and lipid metabolism (Fig. 1b–e, Table 1). We confirmed that MC4R-KO mice showed hepatic steatosis and liver fibrosis after 10 and 20 weeks of WD feeding, respectively (Fig. 1d,g). Under this regime, treatment with anagliptin  had no effect on body weight and adipose tissue weight in MC4R-KO mice (Fig. 1b,c). Although anagliptin treatment was accompanied by slightly decreased liver weight, there was no significant difference in hepatic cholesterol and triglyceride contents (Fig. 1c,d). Anagliptin treatment did not appear to affect systemic glucose and lipid metabolism, whereas it markedly inhibited DPP-4 activity and increased the plasma concentrations of active GLP-1 (Fig. 1e,f, Table 1). In addition, there was a significant reduction in NAFLD activity score (NAS) for MC4R-KO mice subjected to anagliptin treatment for 20 weeks compared to untreated mice (Fig. 1g). Among the three parameters comprising NAS, the scores for “inflammation” and “ballooning degeneration” were significantly decreased by anagliptin treatment. These observations suggest that anagliptin inhibits progression from simple steatosis to NASH in MC4R-KO mice, without affecting systemic glucose and lipid metabolism.

**Table 1:** Effect of anagliptin on serum parameters of MC4R-KO mice fed WD for 10 and 20 weeks.

	10 weeks			20 weeks		
	WT/SD	MC4R-KO/WD	MC4R-KO/WD/Ana	WT/SD	MC4R-KO/WD	MC4R-KO/WD/Ana
AST (U/l)	86.0 ± 8.0	304.5 ± 33.2^##^	270.8 ± 30.0	99.3 ± 7.0	417.3 ± 26.5^##^	395.7 ± 32.3
ALT (U/l)	30.2 ± 0.5	389.1 ± 38.4^#^	457.2 ± 102.8	32.0 ± 2.2	576.7 ± 29.6^##^	596.0 ± 74.1
LDH (U/l)	297.3 ± 27.6	2267.5 ± 245.4^#^	2257.5 ± 643.2	447.7 ± 34.1	2806.3 ± 246.1^##^	2852.3 ± 397.3
TG (mg/dl)	86.5 ± 4.1	184.5 ± 18.9^#^	175.5 ± 21.1	117.2 ± 9.1	95.0 ± 7.5	71.7 ± 4.3*
T-Chol (mg/dl)	58.5 ± 3.0	294.6 ± 16.5^##^	228.1 ± 13.7**	74.3 ± 2.3	253.3 ± 11.0^##^	237.7 ± 13.9

SD, standard diet; WD, Western diet; Ana, anagliptin; TG, triglyceride; T-Chol, total cholesterol.

Date are presented as the mean ± SE. ^#^*p* < 0.05, ^##^*p* < 0.01 between WT/SD and MC4R-KO/WD, *p < 0.05, **p < 0.01 between MC4R-KO/WD and MC4R-KO/WD/Ana. *n* = 6–15.

### Anagliptin inhibits inflammation and fibrosis in the liver of MC4R-KO mice

**Figure 2:**
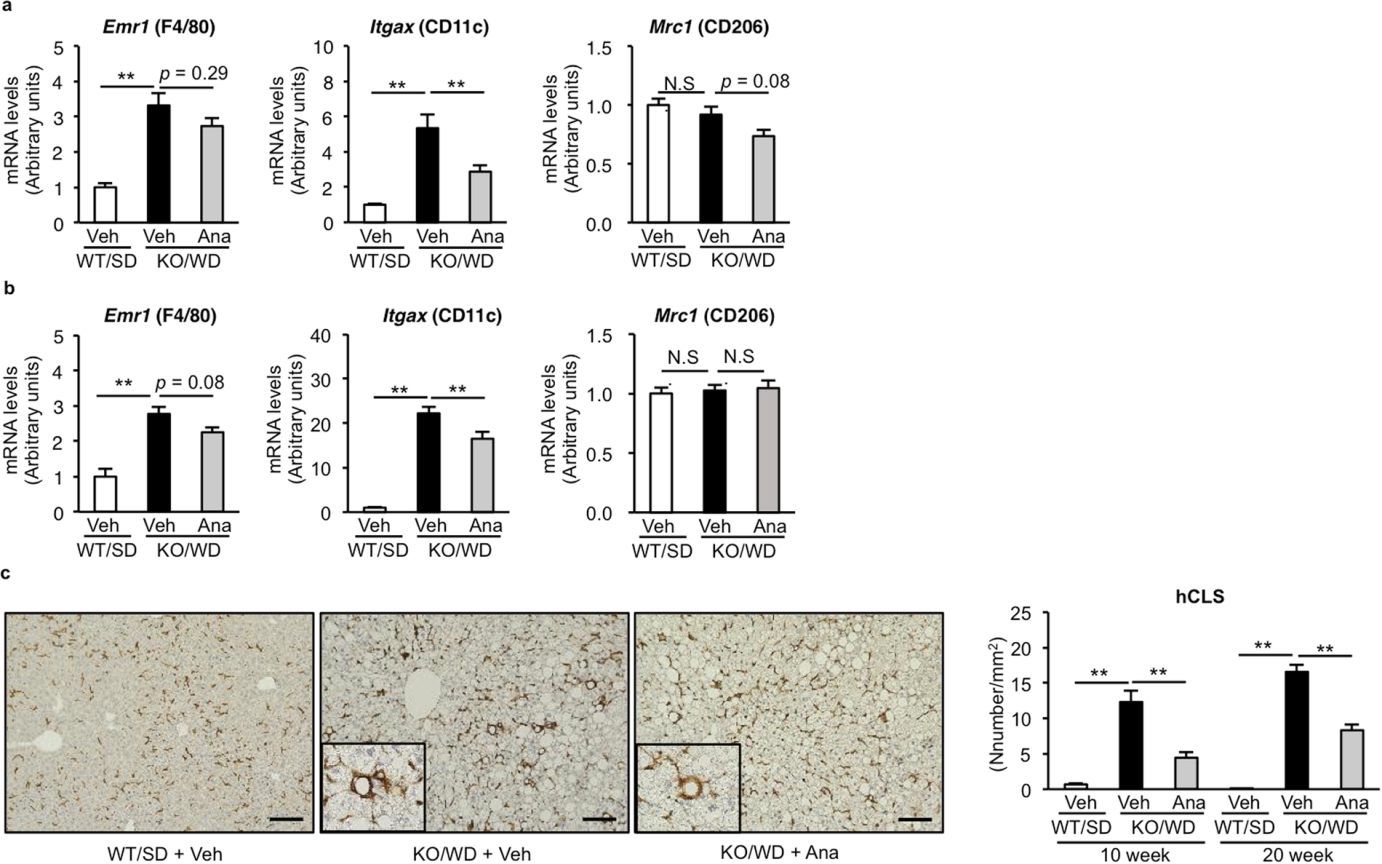
Anagliptin suppresses hepatic inflammation in MC4R-KO mice. Hepatic mRNA expression of inflammation-related genes after 10 **(a)** and 20 **(b)** weeks of treatment with anagliptin. **(c)** F4/80 immunostaining of the liver at 10 weeks. Insets indicate representative images of hepatic crown-like structure (hCLS). Scale bars, 100 µm. **p < 0.01; N.S. not significant. WT/SD treated with vehicle (*n* = 6), KO/WD treated with anagliptin or vehicle (*n* = 12 and 15 at 10 and 20 weeks, respectively).

**Figure 3:**
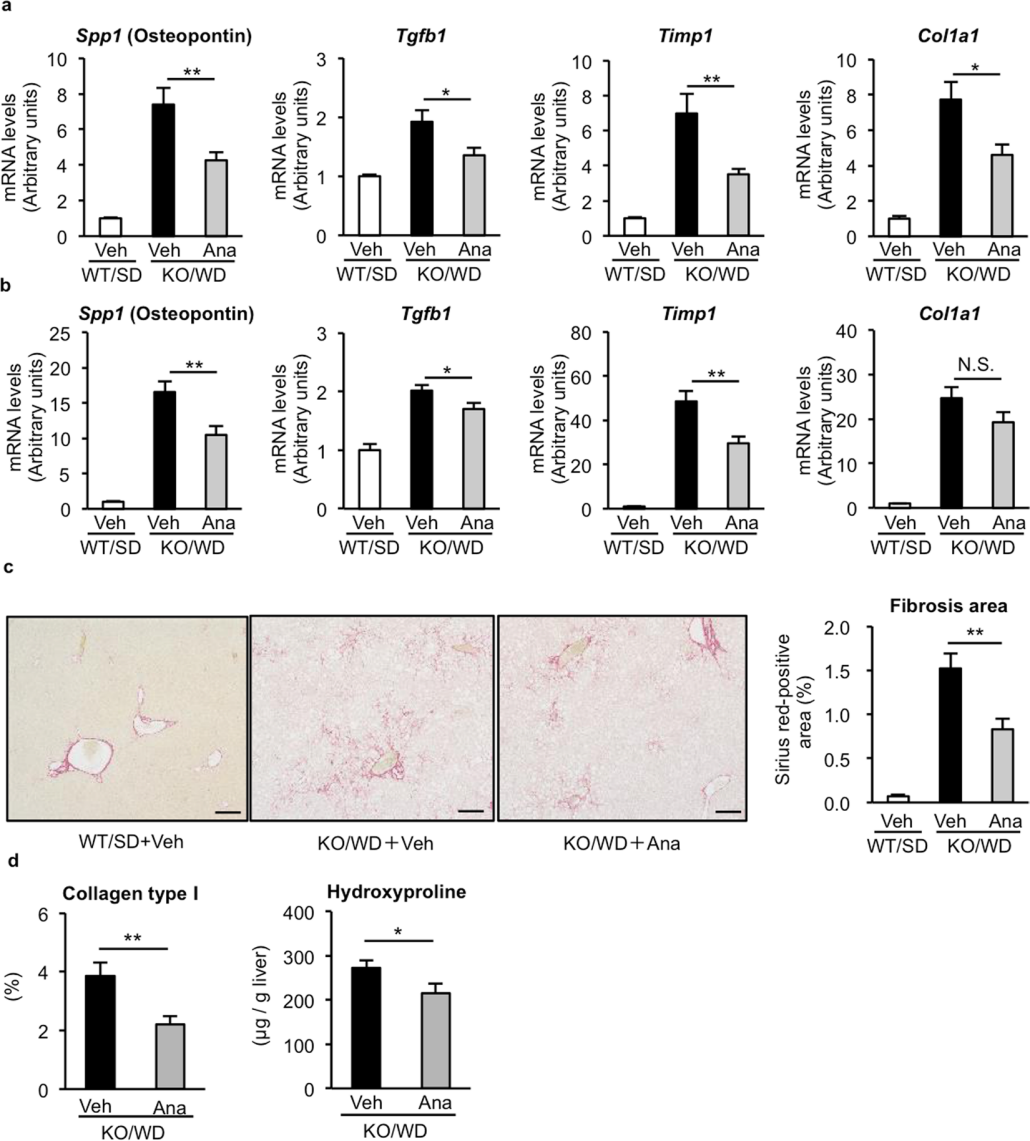
Anagliptin suppresses liver fibrosis in MC4R-KO mice. Hepatic mRNA expression of fibrosis-related genes after 10 **(a)** and 20 **(b)** weeks of treatment with anagliptin. **(c)** Representative images of Sirius Red staining of the liver at 20 weeks. Scale bars, 200 µm. **(d)** Quantification of the collagen type I-positive area and the hydroxyproline content of the liver at 20 weeks. *p < 0.05, **p < 0.01; N.S. not significant. WT/SD treated with vehicle (*n* = 6 at 20 weeks), KO/WD treated with anagliptin or vehicle (*n* = 12 and 15 at 10 and 20 weeks, respectively).

**Figure 4:**
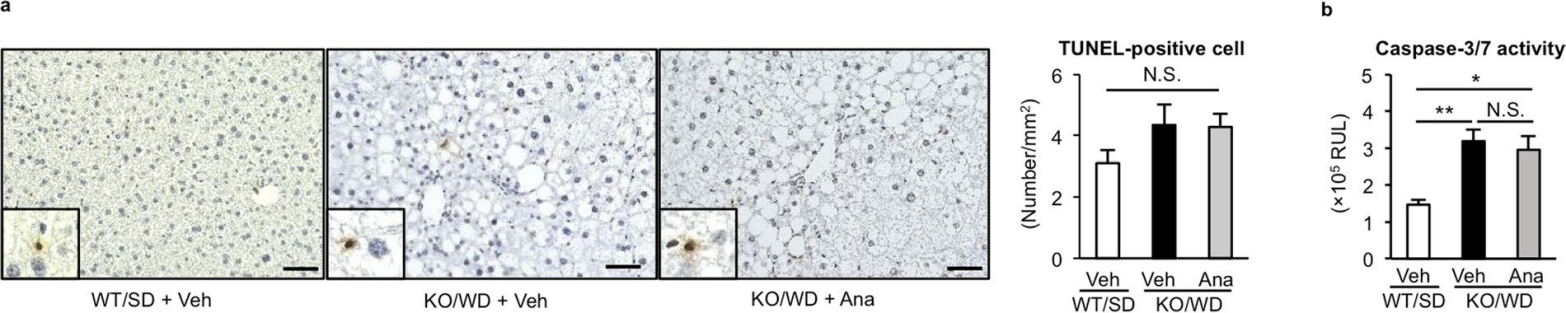
Anagliptin does not affect hepatocyte death in MC4R-KO mice. **(a)** TUNEL staining of the liver after 10-week treatment with anagliptin. Insets indicate representative images of TUNEL-positive cells. Scale bars, 100 µm. **(b)** Caspase-3/7 activity of the liver at 10 weeks. *p < 0.05, **p < 0.01; N.S. not significant (*n* = 6 WT/SD, *n* = 12 KO/WD).

Next, we examined the effect of anagliptin on liver inflammation and fibrosis during NASH development in detail. As previously shown^10^, MC4R-KO mice fed WD showed higher *Emr1* (F4/80, a macrophage marker) and *Itgax* (CD11c, an inflammatory macrophage marker) mRNA levels than wildtype mice fed SD, whereas there was no difference in *Mrc1* (CD206, an anti-inflammatory macrophage marker) mRNA levels at 10 and 20 weeks (Fig. 2a,b). Anagliptin treatment suppressed *Itgax* mRNA expression significantly without changing *Emr1* and *Mrc1* mRNA expression (Fig. 2a,b). MC4R-KO mice fed WD for 10 weeks exhibited hCLS formation, in which CD11c-positive proinflammatory macrophages aggregated around dead hepatocytes, thereby accelerating liver fibrosis^11^. Anagliptin treatment markedly suppressed hCLS formation at 10 and 20 weeks (Fig. 2c). Moreover, anagliptin treatment reduced the extent of the transcription of fibrosis-related genes such as *Spp1* (osteopontin), transforming growth factor-β (*Tgfb1*), tissue inhibitor of metalloproteinase-1 (*Timp1*), and collagen type I (*Col1a1*) (Fig. 3a,b). We verified the preventive effect of anagliptin towards liver fibrosis by means of Sirius Red staining, collagen type I immunostaining, and measurement of hydroxyproline content (Fig. 3c,d). In addition, we examined the effect of anagliptin treatment on cell death in the liver, because the score of ballooning degeneration is positively associated with the number of hCLS in human NASH and hepatocyte death triggers hCLS formation and liver fibrosis in MC4R-KO mice^11,12^. There was no significant difference in the number of TdT mediated dUTP-biotin nick end labeling (TUNEL)-positive cells and caspase-3/7 activity between treatments (Fig. 4), suggesting that anagliptin treatment does not affect overall apoptotic responses in the liver. These observations indicate that anagliptin effectively prevents chronic inflammation and fibrosis in the liver of MC4R-KO mice.

### Anagliptin does not ameliorate adipose tissue inflammation and fibrosis

**Figure 5:**
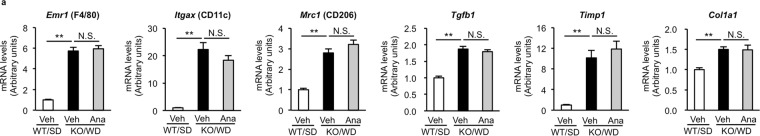
Anagliptin does not affect adipose tissue inflammation in MC4R-KO mice. Adipose tissue mRNA expression of inflammation-related genes after 10-week treatment with anagliptin. **p < 0.01; N.S. not significant (*n* = 6 WT/SD, *n* = 12 KO/WD).

Mounting evidence indicates that obesity-induced inflammation and fibrosis in adipose tissue contribute to ectopic lipid accumulation in the liver^10,15^. DPP-4 inhibitors have also been reported to attenuate adipose tissue inflammation in diet-induced obese mice^16,17^. We therefore determined the effect of anagliptin on obesity-induced chronic inflammation in epididymal fat tissue. Similar to the liver, mRNA expression of genes related to inflammation and fibrosis was significantly increased in the adipose tissue of MC4R-KO mice fed WD compared to wildtype mice fed SD (Fig. 5). However, anagliptin treatment did not suppress the upregulation of these genes (Fig. 5).

### GLP-1 inhibits upregulation of proinflammatory and fibrogenic genes in macrophages

**Figure 6:**
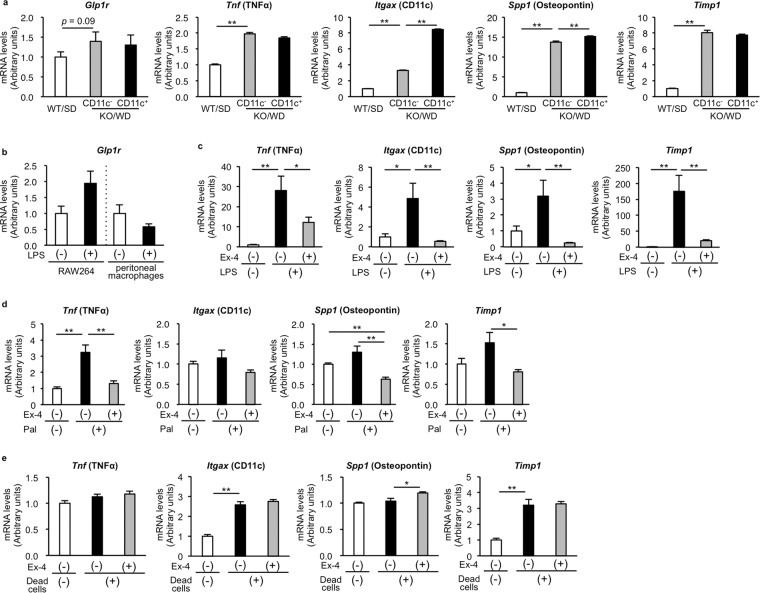
GLP-1 analogue suppresses macrophage inflammatory phenotypes *in vitro*. **(a)** mRNA expression levels of GLP-1 receptor and inflammation- and fibrosis-related genes in hepatic macrophages using microarray database (accession GSE104901). **p < 0.01 (*n* = 5). **(b)** mRNA expression levels of GLP-1 receptor in lipopolysaccharide (LPS)-stimulated macrophages. RAW264 cells or peritoneal macrophages were stimulated with LPS (10 ng/ml) for 6 h. (*n* = 4–6). **(c)** mRNA expression levels of inflammation- and fibrosis-related genes in LPS-stimulated cultured macrophages. RAW264 cells were pretreated with Exendin-4 (Ex-4, 4 ng/ml), a GLP-1 analogue, for 24 h, and then treated with LPS (10 ng/ml) for additional 6 h. *p < 0.05, **p < 0.01 (*n* = 6). **(d)** mRNA expression levels of inflammation- and fibrosis-related genes in palmitate-stimulated cultured macrophages. RAW264 cells were pretreated with Ex-4 (4 ng/ml) for 24 h, and then treated with palmitate (200 µM) for additional 24 h. *p < 0.05, **p < 0.01 (*n* = 4). (**e**) mRNA expression levels of inflammation- and fibrosis-related genes in dead cell-stimulated cultured macrophages. RAW264 cells were pretreated with Ex-4 (4 ng/ml) for 24 h, and then treated with dead hepatocytes (1 × 10^5^ cells/well in 24 well plate) for additional 8 h. *p < 0.05, **p < 0.01 (*n* = 4).

As a molecular mechanism underlying anagliptin action, we focused on macrophages, because anagliptin treatment reduced hCLS formation without affecting hepatic steatosis (Figs. 1 and 2). In addition, anagliptin treatment resulted in a marked increase in the plasma concentrations of active GLP-1 (Fig. 1f). Therefore, we hypothesized that GLP-1 acts on macrophages in the liver to exert anti-inflammatory and anti-fibrogenic effects in MC4R-KO mice. Using microarray analysis reported previously^11^, we first examined mRNA levels in macrophages from normal (WT/SD) and NASH (KO/WD) livers (Fig. 6a). Both CD11c-positive and negative macrophages are derived from NASH livers, in which CD11c-positive macrophages form hCLS and CD11c-negative macrophages scatter in the liver. We confirmed that mRNA levels of inflammation- and fibrosis-related genes were significantly higher in macrophages from KO/WD than in those from WT/SD. In this experiment, mRNA levels of GLP-1 receptor (*Glp1r*) were relatively high in macrophages from KO/WD compared to those from WT/SD. Several lines of evidence indicate the role of Toll-like receptor 4 (TLR4) in the pathogenesis of liver fibrosis or NASH in mice^18,19^. Since RAW264, a macrophage cell line, exhibited upregulation of *Glp1r* mRNAs under inflammatory conditions similar to *in vivo* macrophages from NASH livers (Fig. 6b), we used RAW264 in the following experiments.

In this setting, treatment with Exendin-4, a GLP-1 analogue, markedly suppressed the lipopolysaccharide (LPS)-induced upregulation of the genes related to inflammation and fibrosis (Fig. 6c), whereas anagliptin *per se* did not show such effects (unpublished data). We also examined the effect of palmitate, a representative saturated fatty acid, and dead hepatocytes on RAW264 (Fig. 6d,e). Palmitate plays a major role in obesity-induced metabolic derangements or lipotoxicity, in which palmitate increases proinflammatory cytokine expression in macrophages in TLR4-dependent and independent manners^20^. In this study, Exendin-4 treatment effectively suppressed the palmitate-induced upregulation of the genes related to inflammation and fibrosis in RAW264 (Fig. 6d). On the other hand, no suppressive effects were observed in RAW264 treated with dead hepatocytes (induced by the freeze/thaw treatment) (Fig. 6e). Taken together, these *in vitro* observations are basically consistent with our *in vivo* data (Figs. 2–4) and suggest the involvement of GLP-1 in the anti-inflammatory and anti-fibrotic effects by anagliptin treatment.

### Anagliptin attenuates liver carcinogenesis in MC4R-KO mice

**Figure 7:**
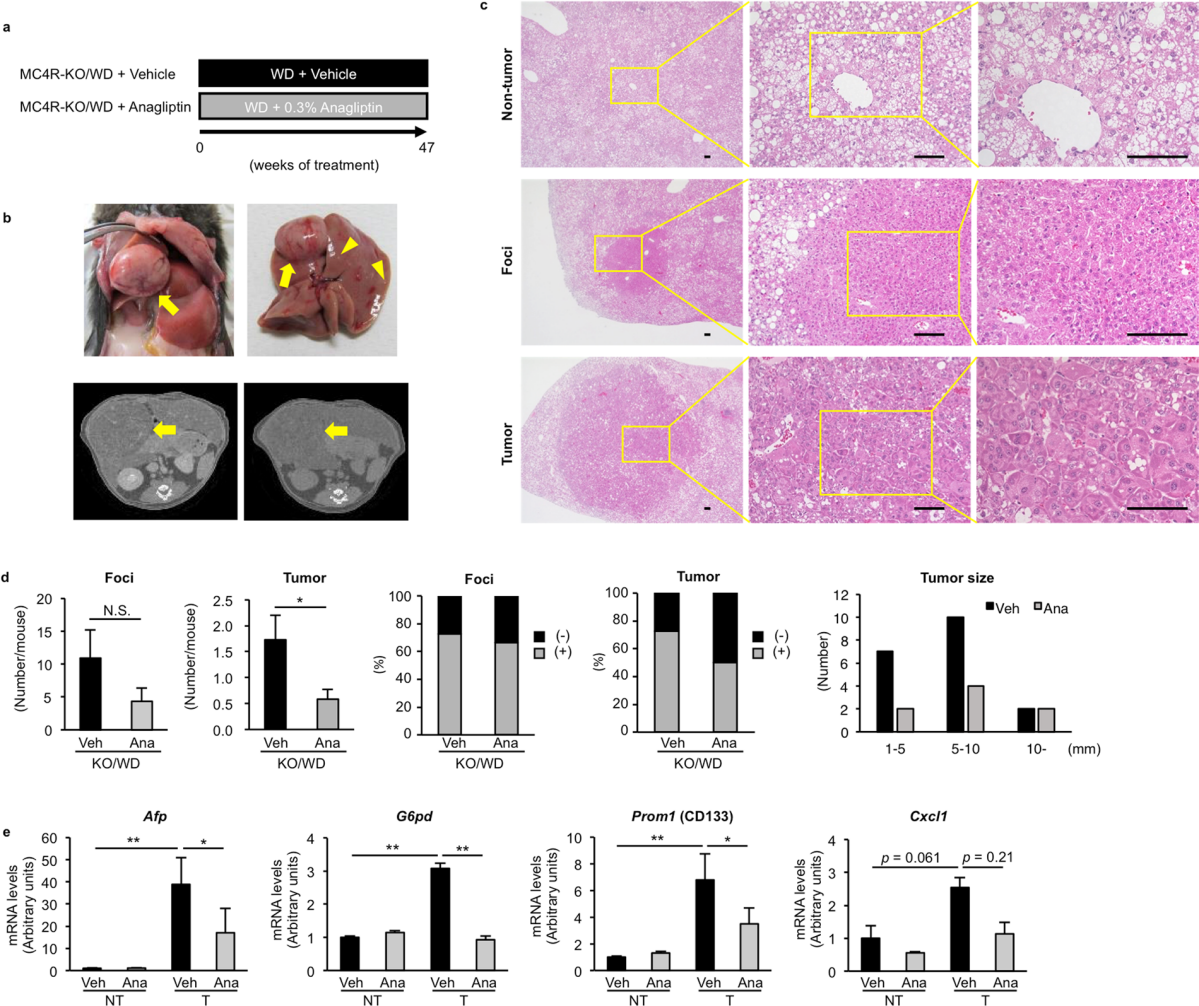
Anagliptin attenuates liver carcinogenesis in MC4R-KO mice. **(a)** Experimental protocol for examination of the preventive effect of anagliptin on the development of liver tumors in MC4R-KO mice. **(b)** Representative images of gross appearance and computed tomography of the liver. Yellow arrows indicate liver tumors and yellow arrowheads indicate preneoplastic foci. **(c)** Representative images of hematoxylin and eosin staining of the liver at 47 weeks. Scale bars, 100 µm. **(d)** Number of foci and tumors and the size of tumors in the liver. *p < 0.05 (Vehicle, *n* = 11; Anagliptin, *n* = 12). **(e)** mRNA expression levels of tumor-related genes in non-tumor (NT) and tumor (T) lesions in the liver. *p < 0.05, **p < 0.01 (*n* = 4).

Finally, we investigated the effect of anagliptin on liver carcinogenesis in MC4R-KO mice (Fig. 7a). Since the sequential development of preneoplastic foci, hyperplastic nodules, hepatocellular adenomas, and hepatocellular carcinomas is known^21,22^, we examined the number of foci and tumors according to their size; the lumps less than 1 mm as foci and the lumps lager than 1 mm as tumors. As previously shown^10^, MC4R-KO mice fed WD showed multiple liver tumors similar to hepatocellular carcinoma in humans (Fig. 7b,c). Microscopic analysis revealed that normal liver architecture was lost, and irregular and thick trabeculae were observed in the tumors. The tumor cells exhibited severe dysplasia, with an increased nuclear-cytoplasmic ratio, enlarged and hyperchromatic nuclei, and fat deposition in the cytoplasm. On the other hand, in the foci, hepatocytes showed the normal shape and the vascular structure was well preserved.

Long-term treatment with anagliptin for 47 weeks reduced the number of liver tumors and tumor size in MC4R-KO mice, whereas there was no apparent difference in the number of foci between the treatments (Fig. 7b–d). We also compared mRNA levels of previously known tumor-associated genes such as *Afp* (α-fetoprotein [AFP]), *G6pd* (glucose-6-phosphate dehydrogenase [G6PD]), *Prom1* (CD133), and *Cxcl1* (chemokine [C-X-C motif] ligand 1 [CXCL1] or keratinocyte-derived chemokine) in tumorous and non-tumorous lesions in the liver of MC4R-KO mice. As a result, mRNA levels of these genes were markedly increased in tumorous lesions compared to non-tumorous lesions (Fig. 7e). We also observed that anagliptin treatment partially prevented the upregulation of these genes (Fig. 7e). We verified that anagliptin treatment did not change serum parameters related to systemic glucose and lipid metabolism at this time point (data not shown). Collectively, these observations indicate that anagliptin effectively prevents the progression from simple steatosis to NASH and the formation of liver tumors.

## Discussion

In this study, we demonstrated that anagliptin treatment effectively prevents the development of inflammation, fibrosis, and tumors in the liver of MC4R-KO mice fed WD. Although growing evidence points to the therapeutic implications of certain types of anti-diabetic agents in NASH^23^, it is technically difficult to distinguish their effects on NASH from their effects on diabetes. For instance, it has been reported that DPP-4 inhibitors ameliorate lipid accumulation and insulin resistance in the liver, along with systemic glucose intolerance^6^. In contrast, anagliptin treatment does not appear to affect serum parameters related to diabetes and dyslipidemia in our NASH model, whereas it markedly inhibits DPP-4 activity and increased active GLP-1 levels in the serum. This is probably because MC4R-KO mice fed WD exhibit morbid obesity with severe insulin resistance and hyperinsulinemia. Indeed, it is known that DPP-4 inhibitors are less effective for treating diabetes in obese patients^24^. Our data also showed that anagliptin treatment does not attenuate hepatic lipid accumulation in MC4R-KO mice fed WD. The multiple parallel hits hypothesis proposes that metabolic stress–induced hepatic inflammation is involved in the progression from hepatic steatosis to NASH^25^. It is, therefore, conceivable that anagliptin affects this process to suppress NASH development. Collectively, this study highlights the glucose metabolism–independent effects of anagliptin on NASH development.

To elucidate the mechanism of action of anagliptin, we focused on hepatic macrophages during the progression from hepatic steatosis to NASH. Using our NASH model, we have previously demonstrated that metabolic stress–induced hepatocyte death triggers hCLS formation. CD11c-positive macrophages surround dead hepatocytes to scavenge their large lipid droplets, thereby accelerating proinflammatory and profibrotic processes^11^. Since CD11c-positive macrophages in hCLS show unique gene expression profiles distinct from CD11c-negative macrophages scattered in the liver of MC4R-KO mice fed WD^11^, it is conceivable that macrophage activation in hCLS is critical to NASH development. In this study, anagliptin treatment effectively inhibits hCLS formation, chronic inflammation, and interstitial fibrosis in the liver without affecting hepatic steatosis. These findings led us to speculate that the mechanism of action of anagliptin lies in hepatocyte death and/or macrophage activation. In this regard, our data indicate the suppressive effect of anagliptin on macrophage activation by increasing circulating GLP-1 levels. This is consistent with previous studies reporting that GLP-1 suppresses production of proinflammatory cytokines and reactive oxygen species in cultured macrophages and obesity-induced chronic inflammation in mice and humans^26–28^. On the other hand, further studies are required to elucidate how anagliptin treatment affects hepatocyte injury or death during the development of NASH. We believe that it will be interesting to determine the effect of GLP-1 analogues on hepatocyte death, hCLS formation, and liver fibrosis in our NASH model.

In adipose tissue of obese mice and humans, there is a unique histological structure termed CLS, which is centered on dead adipocytes^29^. We previously demonstrated that CD11c-positive macrophages in CLS sense danger signals from dead adipocytes, thereby inducing obesity-induced chronic inflammation^15^. In this study, we did not detect significant changes in mRNA expression of inflammation- and fibrosis-related genes in adipose tissue. In contrast, Zhuge *et al*. reported that DPP-4 inhibition attenuates adipose tissue inflammation and systemic insulin resistance in diet-induced obese mice by regulating macrophage polarization^17^. The difference in chemicals used in these studies may explain the inconsistency. Indeed, Zhuge *et al*. reported that linagliptin and sitagliptin ameliorate glucose intolerance to a similar extent in obese mice, whereas linagliptin shows greater suppression of adipose tissue inflammation^17^. Another possibility is the involvement of systemic glucose metabolism in the regulation of adipose tissue inflammation. Moreover, tissue-specific expression of DPP-4 may be taken into consideration. Recently, several lines of evidence indicate that DPP-4 expression in each tissue contributes differently to the activity of circulating DPP-4, glucose metabolism, and chronic inflammation in obese mice^16,30^. Further studies are required to identify the target cells and tissues of each DPP-4 inhibitor.

In addition to typical diabetic complications, such as microvascular and macrovascular complications, increasing attention has been paid to diabetes as a risk of HCC. Hyperglycemia, hyperinsulinemia, insulin resistance, and chronic inflammation have been proposed as underlying causes^31^. Indeed, a number of basic and clinical studies have sought to determine the effect of anti-diabetic agents on the development of HCC under diabetic conditions. For instance, we previously reported that the SGLT2 inhibitor, canagliflozin, suppresses the development of hepatic steatosis, NASH, and HCC, along with glucose intolerance^3^. In this study, we garnered evidence that anagliptin can prevent carcinogenesis in our NASH model in a glucose metabolism–independent manner. DPP-4 inhibitors may prevent carcinogenesis by suppressing liver fibrosis in NASH, since liver fibrosis is the strongest determinant of HCC^32–34^. Certain types of DPP-4 inhibitors are also known to show anti-tumor effects in a xenograft model via immune cells^35,36^. As for clinical relevance, DPP-4 expression in tumor specimens is associated with increased serum DPP-4 activity, more advanced clinical stages, and poor prognosis in HCC patients^35^.

In conclusion, we have demonstrated that anagliptin prevents fibrogenesis and carcinogenesis in the liver of MC4R-KO mice fed WD, but only marginally affecting body weight, systemic glucose and lipid metabolism, and hepatic steatosis. Our data suggest that macrophage activation in hCLS is attenuated by anagliptin treatment during progression from simple steatosis to NASH. This study highlights the glucose metabolism–independent effects of anagliptin on NASH and HCC development.

## Materials and Methods

### Materials

Anagliptin was provided by Sanwa Kagaku Kenkyusho Co., Ltd. (Nagoya, Japan). All reagents were purchased from Sigma (St. Louis, MO) or Nacalai Tesque (Kyoto, Japan) unless otherwise noted.

### Animals

MC4R-KO mice on the C57BL/6 J background were kindly provided by Dr. Joel K. Elmquist (University of Texas Southwestern Medical Center)^37^, and age-matched C57BL/6 J wildtype mice were purchased from CLEA Japan (Tokyo, Japan). The animals were housed in a temperature-, humidity- and light-controlled animal room (12-h light and 12-h dark cycle), and allowed free access to water and SD (CE-2; CLEA Japan). In the first experiment, eight week-old male MC4R-KO mice were fed WD (D12079B; 468 kcal/100 g, 41% energy as fat, 34.0% sucrose, 0.21% cholesterol; Research Diets, New Brunswick, NJ) or 0.3% anagliptin -mixed WD for up to 20 weeks to evaluate the effect of anagliptin on the development of NASH. The dose of anagliptin was determined based on a previous report showing that anagliptin treatment for 10 weeks improved glucose metabolism in diet-induced obese mice^38^. Eight-week-old control male wildtype mice were fed SD throughout the experimental period. In the second experiment, to evaluate the effect of anagliptin on the development of liver tumors, eight week-old male MC4R-KO mice were fed WD with or without anagliptin for 47 weeks. At the end of each experiment, the mice were sacrificed, when fed *ad libitum*, under intraperitoneal pentobarbital anesthesia (30 mg/kg).

### Blood analysis

Concentrations of blood glucose and serum insulin were measured by a blood glucose test meter (Glutest Mint; Sanwa Kagaku Kenkyusho) and Mouse Insulin ELISA Kit (Morinaga Institute of Biological Science, Inc., Kanagawa, Japan), respectively. HbA1c levels were measured by Quo-Lab meter (NIPRO, Osaka, Japan). Plasma DPP-4 activity and active GLP-1 levels were determined as described previously^39^.

### Histological analysis

Liver samples were fixed with neutral-buffered formalin, embedded in paraffin, and cut into 4 μm thick sections that were stained with Hematoxylin and eosin and Sirius red^10^. F4/80-positive macrophages and type I collagen were detected immunohistochemically using an anti-F4/80 antibody (MCA497GA, Bio-Rad Laboratories Inc., Hercules, CA) and an anti-type I collagen antibody (1310-01, SouthernBiotech, Birmingham, AL), respectively. Apoptotic cells were detected by TUNEL assay using an Apop-Tag Plus Peroxidase *In Situ* Apoptosis Detection Kit (Millipore, Billerica, MA). Positive areas for Sirius red or type I collagen was measured using the analysis application of BZ-X710 (KEYENCE, Osaka, Japan). TUNEL-positive cells and hCLS were counted in the whole area of each section and expressed as the mean number/mm^2^. According to the NASH clinical research network scoring system^40^, each score for steatosis, inflammation, and hepatocyte ballooning was evaluated. Fibrosis was staged from zero to three using Sirius red-stained sections. For assessment of tumor development, we measured the number and the size of lumps in the liver; the lumps less than 1 mm as foci and the lumps lager than 1 mm as tumors. All histological evaluations were conducted by two or three investigators with appropriate expertise who had no knowledge of the origin of the slides.

### Caspase-3/7 activity assay

Hepatic caspase-3/7 was measured using Caspase-Glo 3/7 assay (Promega, Madison, WI) as described previously^41^.

### Quantitative real-time PCR

Total RNA was extracted from the liver and cultured cells using Sepasol reagent (Nacalai Tesque). Quantitative real-time PCR was performed with StepOnePlus Real-time PCR System using Fast SYBR Green Master Mix Reagent (Thermo Fisher Scientific, San Jose, CA) as described previously^15^. The primers used in this study are listed in Supplementary Table S1. Levels of mRNA were normalized to those of 36B4 or β-actin mRNA. mRNA levels in hepatic macrophages were determined by microarray database (accession GSE104901)^11^.

### Hepatic lipid and hydroxyproline levels

Total lipids in the liver were extracted with ice-cold 2:1 (vol/vol) chloroform/methanol. Hepatic triglyceride and cholesterol concentrations were determined using triglyceride E-test Wako and cholesterol E-test WAKO (FUJIFILM Wako Pure Chemical Co., Osaka, Japan), respectively. Hepatic hydroxyproline concentrations were determined as follows: the liver was lysed in 2 N NaOH for 15 min at 65 °C and lysate was autoclaved for 20 min. After addition of 6 N HCl, the lysate was autoclaved again and charcoal in 4 N KOH was added. After addition of acetate-citrate buffer (pH 6.5) and centrifugation (15,000 × g) for 10 min, the supernatant was mixed with 0.1 M chloramine-T. After 25 min, Ehrlich’s reagent was added to the mixture and heated at 65 °C for 20 min, then centrifuged (15,000 × g) at 4 °C for 3 min. The hydroxyproline concentrations were evaluated by measuring the absorbance of the supernatant at 550 nm.

### Computed tomographic imaging

According to the manufacturer’s instruction, mice were scanned using a computed tomography scanner, LaTheta LCT-200 (Hitachi-Aloka, Tokyo, Japan).

### Cell culture

RAW264 macrophages (RIKEN BioResource Center, Tsukuba, Japan) were maintained as described previously^42^. After pretreatment with or without Exendin-4 for 16 h, RAW264 cells were stimulated with lipopolysaccharide or dead hepatocytes for additional 6 or 8 h, respectively. Primary hepatocytes were induced to cell death by the freeze/thaw treatment.

### Statistical analysis

Data are presented as the mean ± SE. *p* values < 0.05 were considered as statistically significant. Statistical analysis was performed using analysis of variance followed by Tukey’s post hoc test for comparison between groups. Differences between two groups were compared using unpaired Student *t*-test.

### Study approval

All animal experiments were conducted in accordance with the guidelines for the care and use of laboratory animals of Nagoya University. The protocols were approved by the Animal Care and Use Committee, Research Institute of Environmental Medicine, Nagoya University (approval number 18253).

## Supplementary information

Supplementary Information.

## References

[1] Jojima T (2016). Empagliflozin (an SGLT2 inhibitor), alone or in combination with linagliptin (a DPP-4 inhibitor), prevents steatohepatitis in a novel mouse model of non-alcoholic steatohepatitis and diabetes. Diabetol Metab Syndr.

[2] Seko Y (2018). Efficacy and safety of canagliflozin in type 2 diabetes mellitus patients with biopsy-proven nonalcoholic steatohepatitis classified as stage 1-3 fibrosis. Diabetes Metab Syndr Obes.

[3] Shiba K (2018). Canagliflozin, an SGLT2 inhibitor, attenuates the development of hepatocellular carcinoma in a mouse model of human NASH. Sci. Rep..

[4] Deacon CF, Carr RD, Holst JJ (2008). DPP-4 inhibitor therapy: new directions in the treatment of type 2 diabetes. Front Biosci..

[5] Dicembrini I, Monami M, Mannucci E (2019). Dypeptidylpeptidase-4 inhibitors and the cardiovascular system: How to manage the fil rouge. Nutr Metab Cardiovasc Dis.

[6] Nakamura K (2017). A long-lasting dipeptidyl peptidase-4 inhibitor, teneligliptin, as a preventive drug for the development of hepatic steatosis in high-fructose diet-fed ob/ob mice. Int J Mol Med.

[7] Rohrborn D, Wronkowitz N, Eckel J (2015). DPP4 in Diabetes. Front Immunol.

[8] Miyazaki M (2012). Increased hepatic expression of dipeptidyl peptidase-4 in non-alcoholic fatty liver disease and its association with insulin resistance and glucose metabolism. Mol Med Rep.

[9] Farrell G (2019). Mouse Models of Nonalcoholic Steatohepatitis: Toward Optimization of Their Relevance to Human Nonalcoholic Steatohepatitis. Hepatology.

[10] Itoh M (2011). Melanocortin 4 receptor-deficient mice as a novel mouse model of nonalcoholic steatohepatitis. Am J Pathol.

[11] Itoh, M. *et al*. CD11c+ resident macrophages drive hepatocyte death-triggered liver fibrosis in a murine model of nonalcoholic steatohepatitis. *JCI Insight* **2**, 10.1172/jci.insight.92902 (2017). 10.1172/jci.insight.92902 PMC5752377 29202448

[12] Itoh M (2013). Hepatic crown-like structure: a unique histological feature in non-alcoholic steatohepatitis in mice and humans. PLoS One.

[13] Aroor AR (2015). Dipeptidyl peptidase-4 inhibition ameliorates Western diet-induced hepatic steatosis and insulin resistance through hepatic lipid remodeling and modulation of hepatic mitochondrial function. Diabetes.

[14] Choi SH (2017). Gemigliptin ameliorates Western-diet-induced metabolic syndrome in mice. Can J Physiol Pharmacol.

[15] Tanaka M (2014). Macrophage-inducible C-type lectin underlies obesity-induced adipose tissue fibrosis. Nat Commun.

[16] Ghorpade DS (2018). Hepatocyte-secreted DPP4 in obesity promotes adipose inflammation and insulin resistance. Nature.

[17] Zhuge F (2016). DPP-4 Inhibition by Linagliptin Attenuates Obesity-Related Inflammation and Insulin Resistance by Regulating M1/M2 Macrophage Polarization. Diabetes.

[18] Csak T (2011). Deficiency in myeloid differentiation factor-2 and toll-like receptor 4 expression attenuates nonalcoholic steatohepatitis and fibrosis in mice. Am J Physiol Gastrointest Liver Physiol.

[19] Ye D (2012). Toll-like receptor-4 mediates obesity-induced non-alcoholic steatohepatitis through activation of X-box binding protein-1 in mice. Gut.

[20] Iwasaki Y (2014). Activating transcription factor 4 links metabolic stress to interleukin-6 expression in macrophages. Diabetes.

[21] Bannasch, P., Enzmann, H., Klimek, F., Weber, E. & Zerban, H. Significance of sequential cellular changes inside and outside foci of altered hepatocytes during hepatocarcinogenesis. *Toxicol Pathol* **17**, 617–628; discussion 629, 10.1177/0192623389017004107 (1989). 10.1177/0192623389017004107 2697940

[22] Cast A (2018). C/EBPα-dependent preneoplastic tumor foci are the origin of hepatocellular carcinoma and aggressive pediatric liver cancer. Hepatology.

[23] Snyder HS (2018). Non-alcoholic Fatty Liver Disease: A Review of Anti-diabetic Pharmacologic Therapies. J Clin Transl Hepatol.

[24] Aso Y (2012). Serum level of soluble CD26/dipeptidyl peptidase-4 (DPP-4) predicts the response to sitagliptin, a DPP-4 inhibitor, in patients with type 2 diabetes controlled inadequately by metformin and/or sulfonylurea. Transl Res.

[25] Buzzetti E, Pinzani M, Tsochatzis EA (2016). The multiple-hit pathogenesis of non-alcoholic fatty liver disease (NAFLD). Metabolism.

[26] Salim HM (2017). Teneligliptin, a dipeptidyl peptidase-4 inhibitor, attenuated pro-inflammatory phenotype of perivascular adipose tissue and inhibited atherogenesis in normoglycemic apolipoprotein-E-deficient mice. Vascul Pharmacol.

[27] Lee YS (2012). Glucagon-like peptide-1 inhibits adipose tissue macrophage infiltration and inflammation in an obese mouse model of diabetes. Diabetologia.

[28] He L (2013). Anti-inflammatory effects of exendin-4, a glucagon-like peptide-1 analog, on human peripheral lymphocytes in patients with type 2 diabetes. J Diabetes Investig.

[29] Cinti S (2005). Adipocyte death defines macrophage localization and function in adipose tissue of obese mice and humans. J Lipid Res.

[30] Varin EM (2019). Circulating Levels of Soluble Dipeptidyl Peptidase-4 Are Dissociated from Inflammation and Induced by Enzymatic DPP4 Inhibition. Cell Metab.

[31] Giovannucci E (2010). Diabetes and cancer: a consensus report. CA Cancer J Clin.

[32] Angulo P (2015). Liver Fibrosis, but No Other Histologic Features, Is Associated With Long-term Outcomes of Patients With Nonalcoholic Fatty Liver Disease. Gastroenterology.

[33] Ekstedt M (2015). Fibrosis stage is the strongest predictor for disease-specific mortality in NAFLD after up to 33 years of follow-up. Hepatology.

[34] Vilar-Gomez E (2018). Fibrosis Severity as a Determinant of Cause-Specific Mortality in Patients With Advanced Nonalcoholic Fatty Liver Disease: A Multi-National Cohort Study. Gastroenterology.

[35] Nishina S (2019). Dipeptidyl Peptidase 4 Inhibitors Reduce Hepatocellular Carcinoma by Activating Lymphocyte Chemotaxis in Mice. Cell Mol Gastroenterol Hepatol.

[36] Hollande C (2019). Inhibition of the dipeptidyl peptidase DPP4 (CD26) reveals IL-33-dependent eosinophil-mediated control of tumor growth. Nat Immunol.

[37] Balthasar N (2005). Divergence of melanocortin pathways in the control of food intake and energy expenditure. Cell.

[38] Nakaya K (2013). Dipeptidyl peptidase-4 inhibitor anagliptin ameliorates diabetes in mice with haploinsufficiency of glucokinase on a high-fat diet. Metabolism.

[39] Yamashita S (2019). Contribution of intestinal dipeptidyl peptidase-4 inhibition for incretin-dependent improved glucose tolerance in mice. Eur J Pharmacol.

[40] Juluri R (2011). Generalizability of the nonalcoholic steatohepatitis Clinical Research Network histologic scoring system for nonalcoholic fatty liver disease. J Clin Gastroenterol.

[41] Goto T (2018). Obeticholic acid protects against hepatocyte death and liver fibrosis in a murine model of nonalcoholic steatohepatitis. Sci Rep.

[42] Suganami T, Nishida J, Ogawa Y (2005). A paracrine loop between adipocytes and macrophages aggravates inflammatory changes: role of free fatty acids and tumor necrosis factor α. Arterioscler Thromb Vasc Biol.

