# Supplementary material for: Dipeptidyl peptidase-4 inhibition prevents nonalcoholic steatohepatitis–associated liver fibrosis and tumor development in mice independently of its anti-diabetic effects
Source: Sci Rep. 2020 Jan 22;10:983. doi: 10.1038/s41598-020-57935-6 (PMC6976646; doi:10.1038/s41598-020-57935-6)
Supplement: Supplementary file 1 — Supplementary Information. [file 41598_2020_57935_MOESM1_ESM.pdf]

Dipeptidyl peptidase-4 inhibition prevents nonalcoholic steatohepatitis–associated liver fibrosis and tumor development in mice independently of its anti-diabetic effects

Mitsuhiro Kawakubo[1,3], Miyako Tanaka[1,4*], Kozue Ochi[1], Akiko Watanabe[1], Marie Saka-Tanaka[1,5], Yohei Kanamori[1], Naoki Yoshioka[1,6], Satoko Yamashita[8], Moritaka Goto[8], Michiko Itoh[1,9,10], Ibuki Shirakawa[1], Sayaka Kanai[11], Hiromi Suzuki[2,7], Makoto Sawada[2,7], Ayaka Ito[1,4], Masatoshi Ishigami[6], Mitsuhiro Fujishiro[6], Hiroshi Arima[3], Yoshihiro Ogawa[1,12,13], Takayoshi Suganami[1,4*]

[1] Department of Molecular Medicine and Metabolism, [2] Department of Brain Function, Research Institute of Environmental Medicine, Nagoya University, Nagoya, Japan; [3] Department of Endocrinology and Diabetes, [4] Department of Immunometabolism, [5] Department of Nephrology, [6] Department of Gastroenterology and Hepatology, [7] Department of Molecular Pharmacokinetics, Nagoya University Graduate School of Medicine, Nagoya, Japan; [8] Pharmaceutical Research Laboratories, Sanwa Kagaku Kenkyusho Co., Ltd., Nagoya, Japan; [9] Department of Organ Network and Metabolism, [10] Kanagawa Institute of Industrial Science and Technology, Kawasaki, Japan; [11] Department of Molecular Endocrinology and Metabolism, Graduate School of Medical and Dental Sciences, Tokyo Medical and Dental University, Tokyo, Japan; [12] Department of Medicine and Bioregulatory Science, Graduate School of Medical Sciences, Kyushu University, Fukuoka, Japan; [13] Japan Agency for Medical Research and Development, CREST, Tokyo, Japan.

*Corresponding Author:
Miyako Tanaka, Ph.D. and Takayoshi Suganami, M.D., Ph.D.; Department of Molecular Medicine and Metabolism, Research Institute of Environmental Medicine, Nagoya University, Nagoya, Japan. Tel: +81-52-789-3881, Fax: +81-52-789-5047, E-mail: tanaka@riem.nagoya-u.ac.jp, suganami@riem.nagoya-u.ac.jp.

Supplementary Table 1. Primers used in the present study

Genes	Primers	Primers
Afp	Forward	AGGAGAAATGGTCCGGCTG
	Reverse	GTCCAATGAAAATGTCGGCC
Col1a1	Forward	CCTCAGGGTATTGCTGGACAAC
	Reverse	ACCACTTGATCCAGAAGGACCTT
Cxcl1	Forward	ACTGCACCCAAACCGAAGTC
	Reverse	TGGGGACACCTTTTAGCATCTT
Emr1	Forward	CTTTGGCTATGGGCTTCCAGTC
	Reverse	GCAAGGAGGACAGAGTTTATCGTG
G6pd	Forward	CTGGAACCGCATCATCGTGGAG
	Reverse	CCTGATGATCCCAAATTCATCAAAATAG
Itgax	Forward	GCCATTGAGGGCACAGAGA
	Reverse	GAAGCCCTCCTGGGACATCT
Mrc1	Forward	CGGTGAACCAAATAATTACCAAAAT
	Reverse	GTGGAGCAGGTGTGGGCT
Prom1	Forward	TTATGCCATCACAGAGAAGATGAC
	Reverse	GAACCAGAACAAATTCAGAGGG
Spp1	Forward	CCATCTCAGAAGCAGAATCTCC
	Reverse	ATCGTCATCATCGTCGTCC
Tgfb1	Forward	CCTGAGTGGCTGTCTTTTGACG
	Reverse	AGTGAGCGCTGAATCGAAAGC
Timp1	Forward	CATCACGGCCGCCTA
	Reverse	AAGCTGCAGGCACTGATGTG

Tnfa	Forward	ACCCTCACACTCAGATCATCTTC
	Reverse	TGGTGGTTTGCTACGACGT
36B4	Forward	GGCCCTGCACTCTCGCTTTC
	Reverse	TGCCAGGACGCGCTTGT
β-actin	Forward	GGCCCTGCACTCTCGCTTTC
	Reverse	TGCCAGGACGCGCTTGT
